# Comparison of netupitant/palonosetron with 5-hydroxytryptamine-3 receptor antagonist in preventing of chemotherapy-induced nausea and vomiting in patients undergoing hematopoietic stem cell transplantation

**DOI:** 10.3389/fonc.2023.1280336

**Published:** 2023-11-21

**Authors:** Hang Zhang, Qiang Zeng, Tian Dong, Xinchuan Chen, Pu Kuang, Jian Li, Qiuhui Wu, Ting Liu, Ting Niu, Zhigang Liu, Jie Ji

**Affiliations:** ^1^ Department of Hematology and Institute of Hematology, West China Hospital, Sichuan University, Chengdu, China; ^2^ Stem Cell Transplantation and Cellular Therapy Division, Clinic Trial Center, West China Hospital, Sichuan University, Chengdu, China

**Keywords:** chemotherapy-induced nausea and vomiting, netupitant/palonosetron, hematopoietic stem cell transplantation, 5-hydroxytryptamine-3 receptor antagonist, neurokinin-1 receptor antagonist

## Abstract

**Background:**

The use of 5-hydroxytryptamine-3 receptor antagonists (5HT_3_RA) has long been considered the standard regimen for preventing chemotherapy-induced nausea and vomiting (CINV) prior to hematopoietic stem cell transplantation (HSCT). However, their therapeutic outcomes have been unsatisfactory. NEPA, an oral formulation combining the neurokinin-1 receptor antagonist netupitant and the 5HT_3_RA palonosetron, has received regulatory approval for the management of highly and moderately emetogenic chemotherapy. This study aims to compare the efficacy of NEPA with that of 5HT_3_RA alone in preventing CINV among patients undergoing multiday conditioning chemotherapy prior to HSCT.

**Patients and methods:**

We conducted a retrospective analysis of patients who underwent HSCT between September 2019 and September 2022. Efficacy outcomes were assessed based on the rates of patients achieving complete response (CR: no emesis and no use of rescue medication), complete control (CC: CR without significant nausea), no vomiting, and no significant nausea.

**Results:**

The NEPA group consisted of 106 patients, while the 5HT_3_RA group included 107 patients. The NEPA group exhibited significantly higher rates of CR compared to the 5HT_3_RA group during the overall phase (71.7% vs. 32.7%, P<0.001), acute phase (78.3% vs. 43.0%, P<0.001), and delayed phase (84.9% vs. 58.9%, P<0.001). Similarly, rates of CC, no vomiting, and no significant nausea were significantly better in the NEPA group across all phases (P<0.001).

**Conclusion:**

NEPA demonstrated superior efficacy compared to 5HT_3_RA in preventing CINV during all phases of multiday conditioning regimens among patients undergoing HSCT.

## 1 Introduction

Over the past few decades, hematopoietic stem cell transplantation (HSCT) has emerged as the standard treatment for hematologic malignancies. Patients undergoing HSCT receive high-dose chemotherapy prior to transplantation, and chemotherapy-induced nausea and vomiting (CINV) is one of the most common and distressing adverse events (AEs) during chemotherapy. CINV can have a significant impact on patients’ daily lives, leading to malnutrition, dehydration, electrolyte imbalance, and poor adherence to chemotherapy regimens (1, 2).

Currently, the 5-hydroxytryptamine-3 receptor antagonist (5HT_3_RA) is considered a fundamental component of antiemetic strategies for HSCT conditioning chemotherapy. However, the efficacy of 5HT_3_RA in controlling CINV is unsatisfactory, with reported complete response (CR) rates, indicating the absence of emesis without the use of rescue medication, ranging from 8.3% to 36%. The control rate for nausea is even lower, approximately 8.3% to 26% (3, 4). In recent years, efforts have been made to improve the management of CINV and enhance the patient experience during chemotherapy. Novel antiemetic drugs and CINV management guidelines have shown promising results. One such drug is the neurokinin-1 receptor antagonist (NK1-RA), which was introduced in the 2000s as a potent antiemetic agent that effectively reduces the incidence of CINV (5, 6). Netupitant/palonosetron (NEPA) is a fixed-dose combination of 0.50 mg palonosetron (PALO), a second-generation 5HT_3_RA, and 300 mg netupitant, a highly selective NK1-RA (7, 8). Both netupitant and PALO have long half-lives of 90 hours and 40 hours, respectively. The combination of netupitant and PALO has been proven effective in preventing CINV during both the acute and delayed phases following single-day chemotherapy (9–11). In addition, an *in vitro* study has demonstrated that the combination of netupitant and PALO enhances the inhibition of substance P, a ligand acting on the central pathway, compared to either drug alone (12). This *in vitro* synergy, combined with the long duration of action, has led to the development of NEPA, which is expected to offer clinical advantages in preventing CINV.

Several studies have demonstrated the efficacy and safety of NEPA, with or without dexamethasone (DEX), in preventing CINV in patients undergoing autologous HSCT. Two phase II clinical trials investigated the use of NEPA in patients receiving BEAM (carmustine + etoposide + cytarabine + melphalan) or FEAM (fotemustine + etoposide + cytarabine + melphalan) chemotherapy (13, 14). Additionally, two observational studies described its effectiveness during high-dose melphalan chemotherapy (15, 16). However, these studies primarily focused on specific chemotherapy regimens, which may not adequately represent the diverse and complex conditioning regimens used prior to HSCT, particularly those involving multiday high-dose chemotherapy. The pathophysiology of CINV caused by multiday chemotherapy depends on the emetogenic risk of individual chemotherapy agents and their sequence. Furthermore, acute and delayed CINV can overlap during the course of chemotherapy, making it challenging to recommend a specific antiemetic regimen for each multiday chemotherapy regimen prior to HSCT (6, 17).

Based on the aforementioned studies, we conducted the first case-control study to compare the efficacy of NEPA with 5HT_3_RA alone, without additional DEX, in preventing CINV in patients undergoing HSCT. This study specifically targeted a wider range of conditioning chemotherapy regimens to comprehensively evaluate the effectiveness of NEPA in preventing CINV in this population.

## 2 Patients and methods

### 2.1 Study design and patients

In this study, we included patients who underwent HSCT at West China Hospital between September 2019 and September 2022. The inclusion criteria were as follows: (1) patients aged ≥ 18 years, (2) patients with hematological malignancies receiving highly emetogenic chemotherapy (HEC) or moderately emetogenic chemotherapy (MEC) conditioning regimens prior to HSCT [the emetic risk was assessed based on the National Comprehensive Cancer Network (NCCN) antiemesis guideline (6)], (3) patients receiving either oral NEPA (one dose every 72 hours during conditioning chemotherapy, or only one dose if conditioning was less than 72 hours) or intravenous 5HT_3_RA (one dose every day from day 1 to 2 days after chemotherapy completion) as antiemetic regimen, and (4) patients with an ECOG performance status scale of ≤ 2. The selection of the antiemetic regimen is determined by the patient based on his or her financial capacity. The exclusion criteria were as follows: (1) patients experiencing nausea or vomiting within 12 hours before starting the conditioning regimens, and (2) patients receiving DEX as antiemetic prophylaxis. Patients were assigned to either the NEPA group or the 5HT_3_RA group based on their antiemetic regimens. DEX was administered to every participant (5mg per day) during the study as an anti-inflammatory therapy to mitigate chemotherapy toxicity rather than as an antiemetic prophylaxis. This study was approved by the Biological and Medical Ethics Committee of the West China Hospital, Sichuan University. Because the study involved only de-identified, routinely collected information from the medical record, patient informed consent was deemed not necessary.

### 2.2 Assessment

Stem cell infusion will be performed 2 days after chemotherapy completion. Consequently, our comprehensive observation phase spanned from the start of the chemotherapy administration to 48 hours after chemotherapy completion to avoid the influence of nausea due to dimethyl sulfoxide concomitant with stem cell infusion or intestinal acute graft-versus-host disease. The dietary status, occurrence of nausea, vomiting, and adverse events (dizziness, constipation, headache, and hiccups) was assessed during the acute phase (on the same days as chemotherapy administration), the delayed phase (0-48 hours after chemotherapy completion), and the overall phase (including both the acute and delayed phases) based on nursing records and progress notes. The intensity of nausea was evaluated according to common terminology criteria for adverse events (CTCAE), which comprised four categories: grade 0 (no nausea), grade 1 (mild nausea, loss of appetite without decrease of oral intake), grade 2 (moderate nausea, decreased oral intake without significant weight loss), or grade 3 (severe nausea, inadequate oral intake with requirement of tube feeding or parenteral nutrition). The use of rescue medication, defined as any additional medication taken to alleviate nausea or emesis during the overall phase, was documented according to physician orders.

The primary outcome measure was the rate of CR, defined as the absence of emesis and the absence of rescue medication usage. Secondary outcome measures included: (1) the rate of complete control (CC), defined as CR without significant nausea; (2) the rate of no emesis; (3) the rate of no significant nausea (none or mild nausea); (4) the rate of treatment success, defined as maintenance of CR status during the overall phase; (5) the occurrence of adverse events (including dizziness, constipation, headache, and hiccups); and (6) the average frequency of antiemetic medication per day during the overall phase, including both scheduled and rescue medication.

### 2.3 Statistical analysis

Quantitative variables were presented as medians, with 1st and 3rd quartile values, and were compared between the two groups using the Mann-Whitney U test. Qualitative variables were reported as patient numbers and percentages, and comparisons were made using the chi-squared test. Fisher’s exact test was employed when the expected frequency in any of the cells was below 5. The analysis of treatment success was conducted using the Kaplan-Meier method, considering any patient who experienced emesis or required rescue medication as a treatment failure. Group comparisons were performed using the log-rank test. A two-sided P value of less than 0.05 was considered statistically significant. The statistical analysis and generation of figures were performed using the R programming language (version 4.2.1).

## 3 Results

### 3.1 Baseline characteristics

A total of 213 patients undergoing HSCT were included in our study between September 2019 and September 2022. The NEPA group consisted of 106 patients, while the 5HT_3_RA group comprised 107 patients receiving palonosetron or tropisetron as antiemetic therapy (see **Table 1** for detailed information). Among the participants, approximately 70% received MEC as conditioning regimens, while the remaining patients received HEC. The HEC group included melphalan-based regimens, such as high-dose melphalan (70mg/m2 or 100 mg/m2 d1-2) and BenMel (bendamustine 200 mg/m2 d1-2+ melphalan 70mg/m2 or 100mg/m2 d3-4). The MEC group included busulfan-based regimens, including BFA (busulfan 3.2mg/kg d1-4+ fludarabine35mg/m2 d1-5 + cytarabine 1g/m2 d1-5) and GCB (busulfan 3.2mg/kg d1-4 + gemcitabine 2.5g/m2 d1,5 + cladribine 6 mg/m2 d1-5). In this study, patients who underwent 2-day chemotherapy were administered a single dose of NEPA, whereas patients undergoing 4- or 5-day of chemotherapy received a total of two doses of NEPA.

**Table 1:** Patient baseline and chemotherapy characteristics.

	NEPA	5-HT_3_RA
	(n=106)	(n=107)
Age/Median(1st-3rd quartile)	50.5(39-56)	49(35-55)
Sex(M/F)	46/60	47/60
Diagnosis		
Leukemia	36(34.0%)	27(25.2%)
Lymphoma	32(30.2%)	36(33.6%)
Plasma cell dyscrasias	32(30.2%)	33(30.8%)
Myelodysplastic syndrome	6(5.7%)	11(10.3%)
Type of HSCT		
Autologous HSCT	64(60.4%)	69(64.5%)
Allogeneic HSCT	42(39.6%)	38(35.5%)
Type of chemotherapy		
HEC (Melphalan-based) ^a^	32(30.2%)	33(30.8%)
MEC (Busulfan-based) ^b^	74(69.8%)	74(69.2%)
Chemotherapy duration		
2d	11(10.4%)	21(19.6%)
4d	21(19.8%)	12(11.2%)
5d	74(69.8%)	74(69.2%)
Type of 5HT_3_RA		
Palonosetron ^c^	–	43(40.2%)
Tropisetron ^d^	–	64(59.8%)

^a^melphalan-based regimens: high-dose melphalan (≥ 140mg/m^2^), and BenMel (melphalan ≥ 140mg/m^2^ + bendamustine).

^b^busulfan-based regimens: BFA (busulfan + fludarabine + cytarabine), and GCB (busulfan + gemcitabine + cladribine).

^c^0.25mg every day.

^d^5mg every day.

### 3.2 Efficacy in the NEPA group and 5HT_3_RA group

**Figure 1:**
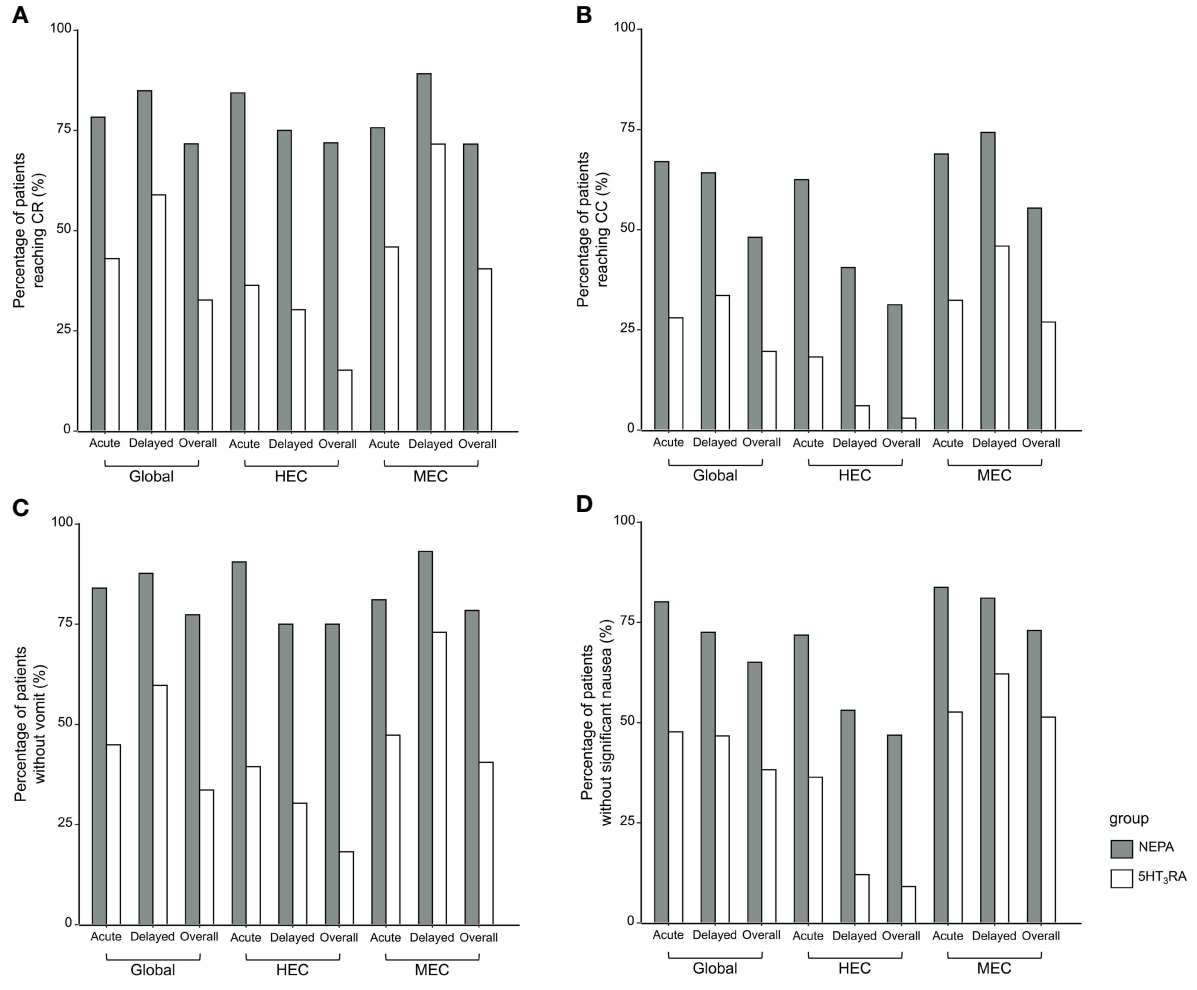
Efficacy outcomes. The histograms show the outcomes for the acute, delayed, and overall phases classified as Global, HEC, and MEC populations. **(A)** Percentage of patients reaching CR **(B)** Percentage of patients reaching CC **(C)** Percentage of patients without vomit **(D)** Percentage of patients without significant nausea.

The efficacy outcomes are presented in **Figure 1**. In the global study population (detailed in **Supplementary Table 1**), the CR rate during the overall phase was 71.7% (76/106) in the NEPA group, significantly higher than the 32.7% (35/107) observed in the 5HT_3_RA group (P < 0.001). Similarly, significant differences in CR rates were observed in both the acute phase (78.3% vs. 43.0%, P < 0.001) and the delayed phase (84.9% vs. 58.9%, P < 0.001). The rate of CC was also significantly higher in the NEPA group compared to the 5HT_3_RA group across all three phases (48.1% vs. 19.6%, P < 0.001 in the overall phase, 67.0% vs. 28.0%, P < 0.001 in the acute phase, 64.2% vs. 33.6%, P < 0.001 in the delayed phase). Furthermore, the NEPA group demonstrated higher rates of no emesis and no significant nausea during all three phases compared to the 5HT_3_RA group.

The frequency of antiemetic medication was also calculated for each group (**Table 2**). The median average daily antiemetic medication during the overall phase was significantly lower in the NEPA group (median: 0.29, 1st-3rd quartile: 0.29-0.33) compared to the 5HT_3_RA group (median: 1.14, 1st-3rd quartile: 1.00-1.29, P < 0.001).

**Table 2:** Adverse events and average frequency of antiemetic medication in the global population.

	NEPA	5HT_3_RA	P Value
	(n=106)	(n=107)	
Adverse events			
Dizziness	37(34.9%)	46(43.0%)	0.285^a^
Constipation	3(2.8%)	4(3.7%)	1.000^b^
Headache	2(1.9%)	2(1.9%)	1.000^b^
Hiccups	0(0.0%)	2(1.9%)	0.498^b^
The average frequency of antiemetic medication/ Median (1^st^-3^rd^ quartile)	0.29 (0.29-0.33)	1.14 (1.00-1.29)	<0.001^c^

^a^Pearson’s Chi-squared test.

^b^Fisher’s exact test.

^c^Mann‐Whitney U test.

In the NEPA group, there were no significant differences in CR rates between the HEC population and the MEC population during all phases (overall: 71.9% vs. 71.6%, p = 1.000; acute: 84.4% vs. 75.7%, p = 0.459; delayed: 75.0% vs. 89.2%, p = 0.115, as shown in **Table 3**). However, CC rates differed between the HEC and MEC populations in the delayed phase (40.6% vs. 74.3%, p = 0.002) and the overall phase (31.3% vs. 55.4%, p = 0.038), while no significant difference was observed in the acute phase (62.5% vs. 68.9%, p = 0.674). In the 5HT_3_RA group, there were no significant differences in all outcomes between the HEC population and the MEC population during acute phase. While all outcomes differed in the delayed and overall phases (as shown in **Table 3**).

**Table 3:** Comparison of the efficacy of NEPA and 5-HT_3_RA in patients receiving HEC and MEC.

	NEPA			5-HT_3_RA		
	HEC (n=32)	MEC (n=74)	P ^a^	HEC (n=33)	MEC (n=74)	P ^a^
Chemotherapy duration						
2d	11(34.4%)	0(0.0%)	–	21(63.6%)	0(0.0%)	–
4d	21(65.6%)	0(0.0%)	–	12(36.4%)	0(0.0%)	–
5d	0(0.0%)	74(100.0%)	–	0(0.0%)	74(100.0%)	–
Complete Response						
acute	27(84.4%)	56(75.7%)	0.459	12(36.4%)	34(45.9%)	0.476
delay	24(75.0%)	66(89.2%)	0.115	10(30.3%)	53(71.6%)	<0.001
overall	23(71.9%)	53(71.6%)	1.000	5(15.2%)	30(40.5%)	0.018
Complete Control						
acute	20(62.5%)	51(68.9%)	0.674	6(18.2%)	24(32.4%)	0.200
delay	13(40.6%)	55(74.3%)	0.002	2(6.1%)	34(45.9%)	<0.001
overall	10(31.3%)	41(55.4%)	0.038	1(3.0%)	20(27.0%)	0.009
No emesis						
acute	29(90.6%)	60(81.1%)	0.347	13(39.4%)	35(47.3%)	0.583
delay	24(75.0%)	69(93.2%)	0.021	10(30.3%)	54(73.0%)	<0.001
overall	24(75.0%)	58(78.4%)	0.898	6(18.2%)	30(40.5%)	0.041
No significant nausea						
acute	23(71.9%)	62(83.8%)	0.252	12(36.4%)	39(52.7%)	0.176
delay	17(53.1%)	60(81.1%)	0.006	4(12.1%)	46(62.2%)	<0.001
overall	15(46.9%)	54(73.0%)	0.018	3(9.1%)	38(51.4%)	<0.001

^a^Pearson’s Chi-squared test.

### 3.3 Subgroup analysis in the NEPA group and 5HT_3_RA group

In the HEC population (**Figure 1**, **Supplementary Table 2**), a significantly higher proportion of patients in the NEPA group achieved CR compared to the 5HT_3_RA group (overall: 71.9% vs. 15.2%, p < 0.001; acute: 84.4% vs. 36.4%, p < 0.001; delayed: 75.0% vs. 30.3%, p < 0.001). Similarly, the rate of CC was significantly higher in the NEPA group (overall: 31.3% vs. 3.0%, p = 0.007; acute: 62.5% vs. 18.2%, p < 0.001; delayed: 40.6% vs. 6.1%, p = 0.003).

In the MEC population (**Figure 1**, **Supplementary Table 3**), patients in the NEPA group were more likely to achieve CR compared to the 5HT_3_RA group (overall: 71.6% vs. 40.5%, p < 0.001; acute: 75.7% vs. 45.9%, p = 0.013; delayed: 89.2% vs. 71.6%, p < 0.001). The CC rate was also significantly higher in the NEPA group (overall: 55.4% vs. 27.0%, p < 0.001; acute: 68.9% vs. 32.4%, p < 0.001; delayed: 74.3% vs. 45.9%, p < 0.001). The NEPA group demonstrated favorable response rates for other outcomes such as CC, no emesis, and no significant nausea during all three phases in both the HEC and MEC populations, and the differences between the two groups were statistically significant.

### 3.4 Antiemetic treatment success analysis

**Figure 2:**
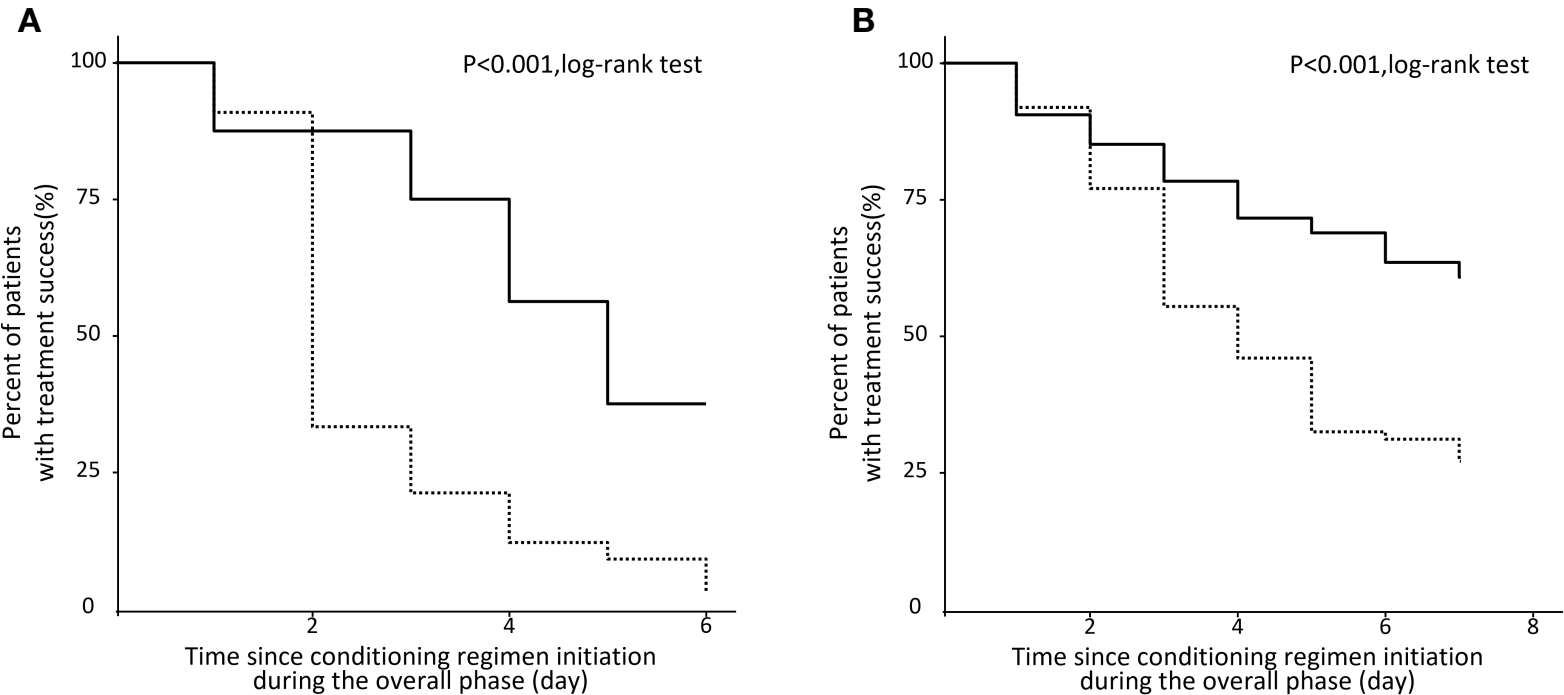
Kaplan‐Meier analysis of patients with no CINV events during the overall phase. **(A)** HEC population. **(B)** MEC population.

The proportion of treatment success (**Figure 2**) during the overall phase was significantly higher in the NEPA group compared to the 5HT_3_RA group in both the HEC population (37.5% vs. 3.0%, p < 0.001) and the MEC population (55.4% vs. 27.0%, p < 0.001).

### 3.5 Safety in the NEPA group and 5HT_3_RA group

Both NEPA and 5HT_3_RA were found to be safe in the management of CINV, as indicated in **Table 2**. None of the patients in our study discontinued participation due to serious AEs. The most commonly reported AE, irrespective of the treatment group (NEPA vs. 5HT_3_RA), was dizziness (34.9% vs. 43.0%, p=0.285). The incidence of AEs did not significantly differ between the two groups.

## 4 Discussion

In our study, we found that the antiemetic regimen of NEPA every 72 hours as an antiemetic regimen provided excellent control of CINV in patients undergoing multiday conditioning regimens before HSCT. The CR rate during the overall phase reached 71.7%, indicating effective CINV control without severe AEs. Furthermore, the NEPA group demonstrated significantly higher efficacy than the 5HT_3_RA group in terms of the rates of CR, CC, no emesis, and no significant nausea across all phases. Subgroup analysis yielded similar findings. These results suggest that NEPA administered every 72 hours is a superior antiemetic regimen for patients undergoing HSCT compared to 5HT_3_RA.

Additionally, two phase II clinical trials have investigated the feasibility of multidose NEPA in patients receiving conditioning chemotherapy regimens such as BEAM or FEAM. The findings from both studies indicated that the majority of patients did not experience emesis during both the acute and delayed phases (13, 14). Another observational study reported the effectiveness of multidose NEPA during high-dose melphalan chemotherapy, with an impressive 93.3% of patients achieving CR (15). It is worth noting that the aforementioned studies utilized a three-dose regimen of NEPA. In contrast, in our study, NEPA was administered once every 72 hours during chemotherapy administration, which meant using a single dose for a two-day regimen and two doses for a four to five-day regimen. This adjustment in dosing was primarily due to the consideration that Netupitant is a moderate inhibitor of cytochrome P450 3A4 (CYP3A4), which may potentially interact with the pharmacokinetics of other drugs metabolized by CYP3A4 (18). This interaction is particularly relevant in the case of busulfan, which is commonly used in HSCT and metabolized by the same enzyme (19). In our study, the multiple doses of NEPA were well-tolerated, and no serious AEs were observed. Therefore, the use of NEPA in conjunction with busulfan can be considered safe.

Delayed CINV, particularly delayed nausea, remains a persistent problem despite advancements in emetic control. Nausea has a more significant impact on patients’ quality of life compared to vomiting, and patients often rank nausea as the most feared adverse event associated with chemotherapy (1, 20). A real-world study evaluating the efficacy of a prophylactic antiemetic regimen consisting of PALO + aprepitant/fosaprepitant + DEX indicated suboptimal control, particularly for delayed nausea (21). However, in our study, NEPA demonstrated significant control over nausea throughout all phases of chemotherapy. Specifically, 65.1% of patients in the NEPA group reported no significant nausea during the overall phase, compared to only 38.3% in the 5HT_3_RA group (P<0.001). NEPA also exhibited superior results during both the acute phase (80.2% vs. 47.7%, P<0.001) and the delayed phase (72.6% vs. 46.7%, P <0.001). Subgroup analyses conducted in the HEC and MEC populations further supported these findings. However, it is worth noting that NEPA’s control of nausea in the HEC population was not as effective as in the MEC population during the delayed and overall phases, highlighting the need for further research on nausea control, particularly during the delayed phase. Similarly, Recent studies have shown the superiority of NEPA in controlling nausea. For instance, a randomized phase III study reported a nonsignificant nausea rate of 78.2% during the delayed phase in patients receiving single-day HEC (22). Furthermore, a real-world study demonstrated the efficacy of NEPA (administered once every other day) in controlling nausea, with rates of no significant nausea slightly higher than our study (acute phase: 78.8%, delayed phase: 74.8%, overall phase: 66.9%) (23).

NEPA is an oral fixed-dose antiemetic with a long half-life, making it a simpler option for administration. Our study demonstrated that NEPA can be given every 72 hours without compromising its efficacy, which simplifies the dosing schedule compared to daily dosing regimens. Additionally, the NEPA group exhibited a lower average frequency of antiemetic medication per day during the overall phase, even when considering rescue medication, which reduces the nursing workload. This finding highlights the practical benefits of NEPA administration. Previous studies have shown that the complexity of administration schedules involving aprepitant has resulted in low adherence to antiemetic guidelines (23). A study by Dranitsaris et al. found that in HEC settings, only 12% of patients received antiemetic prophylaxis with a combination of 5HT_3_RA + NK1-RA + DEX as recommended by the NCCN guidelines (24). Similar low adherence to antiemetic guidelines has been reported in Europe and the US (25, 26). The complexity of NK1-RA-based administration schedules contributes to inconsistent adherence to guidelines and noncompliance by patients (27, 28). In contrast, the simplicity of NEPA administration schedule facilitates easier implementation in real-world clinical settings.

While our study has demonstrated the efficacy and safety of NEPA in managing CINV across diverse conditioning chemotherapy regimens before HSCT, it does have certain limitations. Firstly, it is a retrospective study, which may introduce bias in the collection of relevant data. Secondly, the sample size was relatively small and insufficient for evaluating the efficacy of NEPA in each specific conditioning regimen. In the future, we plan to conduct a larger-scale prospective study to assess the efficacy of NEPA in relation to each chemotherapy regimen, providing more robust evidence in support of its use.

## 5 Conclusions

In conclusion, our study demonstrates that administering NEPA every 72 hours, without additional DEX, is a safe and superior antiemetic regimen compared to a regimen of 5HT_3_RA in patients undergoing multiday conditioning regimens before HSCT. NEPA effectively prevents emesis, particularly nausea, throughout all phases of chemotherapy. However, further investigation is warranted through a randomized controlled clinical trial with a larger sample size to evaluate the efficacy of NEPA in different conditioning regimens for HSCT.

## Data availability statement

The original contributions presented in the study are included in the article/**Supplementary Material**. Further inquiries can be directed to the corresponding authors.

## Ethics statement

The studies involving humans were approved by the Biological and Medical Ethics Committee of the West China Hospital, Sichuan University. The studies were conducted in accordance with the local legislation and institutional requirements. The participants provided their written informed consent to participate in this study.

## Author contributions

HZ: Conceptualization, Data curation, Formal analysis, Methodology, Writing – review & editing, Investigation, Resources, Software, Validation, Visualization, Writing – original draft. QZ: Conceptualization, Data curation, Formal analysis, Investigation, Methodology, Resources, Software, Writing – original draft, Writing – review & editing. TD: Data curation, Investigation, Resources, Writing – review & editing. XC: Data curation, Investigation, Resources, Writing – review & editing. PK: Data curation, Investigation, Resources, Writing – review & editing. JL: Data curation, Investigation, Resources, Writing – review & editing. QW: Data curation, Investigation, Resources, Writing – review & editing. TL: Data curation, Investigation, Resources, Writing – review & editing, Supervision. TN: Data curation, Investigation, Resources, Supervision, Writing – review & editing. ZL: Data curation, Investigation, Resources, Supervision, Writing – review & editing, Conceptualization, Formal analysis, Methodology, Project administration, Validation. JJ: Conceptualization, Data curation, Formal analysis, Methodology, Project administration, Supervision, Writing – review & editing, Funding acquisition.
