# Supplementary material for: Comparison of netupitant/palonosetron with 5-hydroxytryptamine-3 receptor antagonist in preventing of chemotherapy-induced nausea and vomiting in patients undergoing hematopoietic stem cell transplantation
Source: Front Oncol. 2023 Nov 21;13:1280336. doi: 10.3389/fonc.2023.1280336 (PMC10703172; doi:10.3389/fonc.2023.1280336)
Supplement: Supplementary file 1 [file Table_1.docx]

Supplementary Material

| Supplementary table 1. CINV outcomes and adverse effects in the global population | | | |
| --- | --- | --- | --- |
|  | NEPA | 5-HT3 RA | P Value |
|  | (n=106) | (n=107) |  |
| age/Median(min-max) | 50(15-68) | 49(14-65) | 0.263^a^ |
| sex(M/F) | 46/60 | 47/60 | 1.000^b^ |
| Complete Response |  |  |  |
| acute | 83(78.3%) | 46(43.0%) | <0.001^b^ |
| delay | 90(84.9%) | 63(58.9%) | <0.001^b^ |
| overall | 76(71.7%) | 35(32.7%) | <0.001^b^ |
| Complete Control |  |  |  |
| acute | 71(67.0%) | 30(28.0%) | <0.001^b^ |
| delay | 68(64.2%) | 36(33.6%) | <0.001^b^ |
| overall | 51(48.1%) | 21(19.6%) | <0.001^b^ |
| No emesis |  |  |  |
| acute | 89(84.0%) | 48(44.9%) | <0.001^b^ |
| delay | 93(87.7%) | 64(59.8%) | <0.001^b^ |
| overall | 82(77.4%) | 36(33.6%) | <0.001^b^ |
| No significant nausea |  |  |  |
| acute | 85(80.2%) | 51(47.7%) | <0.001^b^ |
| delay | 77(72.6%) | 50(46.7%) | <0.001^b^ |
| overall | 69(65.1%) | 41(38.3%) | <0.001^b^ |
| Adverse events |  |  |  |
| Dizziness | 37(34.9%) | 46(43.0%) | 0.285^b^ |
| Constipation | 3(2.8%) | 4(3.7%) | 1.000^c^ |
| Headache | 2(1.9%) | 2(1.9%) | 1.000^c^ |
| Weakness | 70(66.0%) | 89(83.2%) | 0.007^b^ |
| Hiccups | 0(0.0%) | 2(1.9%) | 0.498^c^ |

a: Wilcoxon rank sum test; b: Pearson's Chi-squared test; c: Fisher's exact test.

| Supplementary table 2. CINV outcomes and adverse effects in the HEC population | | | |
| --- | --- | --- | --- |
|  | NEPA | 5-HT3 RA | P Value |
|  | (n=32) | (n=33) |  |
| age/Median(min-max) | 54(17-67) | 52(14-65) | 0.482^a^ |
| sex(M/F) | 10/22 | 15/18 | 0.357^b^ |
| Complete Response |  |  |  |
| acute | 27(84.4%) | 12(36.4%) | <0.001^b^ |
| delay | 24(75.0%) | 10(30.3%) | <0.001^b^ |
| overall | 23(71.9%) | 5(15.2%) | <0.001^b^ |
| Complete Control |  |  |  |
| acute | 20(62.5%) | 6(18.2%) | <0.001^b^ |
| delay | 13(40.6%) | 2(6.1%) | 0.003^b^ |
| overall | 10(31.3%) | 1(3.0%) | 0.007^b^ |
| No emesis |  |  |  |
| acute | 29(90.6%) | 13(39.4%) | <0.001^b^ |
| delay | 24(75.0%) | 10(30.3%) | <0.001^b^ |
| overall | 24(75.0%) | 6(18.2%) | <0.001^b^ |
| No significant nausea |  |  |  |
| acute | 23(71.9%) | 12(36.4%) | 0.009^b^ |
| delay | 17(53.1%) | 4(12.1%) | 0.001^b^ |
| overall | 15(46.9%) | 3(9.1%) | 0.002^b^ |
| Adverse events |  |  |  |
| Dizziness | 11(34.4%) | 10(30.3%) | 0.932^b^ |
| Constipation | 0(0.0%) | 0(0.0%) | - |
| Headache | 2(6.3%) | 0(0.0%) | 0.239^c^ |
| Weakness | 20(62.5%) | 26(78.8%) | 0.242^b^ |
| Hiccups | 0(0.0%) | 0(0.0%) | - |

a: Wilcoxon rank sum test; b: Pearson's Chi-squared test; c: Fisher's exact test.

| Supplementary table 3. CINV outcomes and adverse effects in the MEC population | | | |
| --- | --- | --- | --- |
|  | NEPA | 5-HT3 RA | P Value |
|  | (n=74) | (n=74) |  |
| age/Median(min-max) | 47(15-68) | 44.5(15-65) | 0.239^a^ |
| sex(M/F) | 36/38 | 32/42 | 0.621^b^ |
| Complete Response |  |  |  |
| acute | 56(75.7%) | 34(45.9%) | <0.001^b^ |
| delay | 66(89.2%) | 53(71.6%) | 0.013^b^ |
| overall | 53(71.6%) | 30(40.5%) | <0.001^b^ |
| Complete Control |  |  |  |
| acute | 51(68.9%) | 24(32.4%) | <0.001^b^ |
| delay | 55(74.3%) | 34(45.9%) | <0.001^b^ |
| overall | 41(55.4%) | 20(27.0%) | <0.001^b^ |
| No emesis |  |  |  |
| acute | 60(81.1%) | 35(47.3%) | <0.001^b^ |
| delay | 69(93.2%) | 54(73.0%) | 0.002^b^ |
| overall | 58(78.4%) | 30(40.5%) | <0.001^b^ |
| No significant nausea |  |  |  |
| acute | 62(83.8%) | 39(52.7%) | <0.001^b^ |
| delay | 60(81.1%) | 46(62.2%) | 0.018^b^ |
| overall | 54(73.0%) | 38(51.4%) | 0.011^b^ |
| Adverse events |  |  |  |
| Dizziness | 26(35.1%) | 36(48.6%) | 0.134^b^ |
| Constipation | 3(4.1%) | 4(5.4%) | 1.000^c^ |
| Headache | 0(0.0%) | 2(2.7%) | 0.497^c^ |
| Weakness | 50(67.6%) | 63(85.1%) | 0.020^b^ |
| Hiccups | 0(0.0%) | 2(2.7%) | 0.497^c^ |

a: Wilcoxon rank sum test; b: Pearson's Chi-squared test; c: Fisher's exact test.
